# Postoperative liver injury after sevoflurane or propofol anesthesia in patients undergoing non-cardiac surgery: a retrospective cohort study

**DOI:** 10.1038/s41598-024-61799-5

**Published:** 2024-05-16

**Authors:** Dae Kyun Ryu, MiHye Park, Seunghyeon Woo, Hyun Seong Cho, Jeong-Jin Min

**Affiliations:** Department of Anesthesiology and Pain Medicine, Samsung Medical Center, Sungkyunkwan University School of Medicine, Seoul, South Korea

**Keywords:** Inhalational anesthetics, Liver injury, Non-cardiac surgery, Propofol, Sevoflurane, Hepatitis, Liver diseases, Outcomes research

## Abstract

Although sevoflurane is generally considered safe, reports suggest that sevoflurane may cause postoperative liver injury more frequently than previously believed. Therefore, we aimed to compare the incidence of clinically significant postoperative liver injury following non-cardiac surgery between patients who underwent sevoflurane anesthesia and propofol-based total intravenous anesthesia. We retrospectively reviewed adult surgical patients from January 2010 to September 2022 who underwent general anesthesia in our center using sevoflurane or propofol over 3 h. After 1:1 propensity score matching, the incidence of postoperative liver injury was compared between the two groups. Out of 58,300 patients reviewed, 44,345 patients were included in the analysis. After propensity score matching, 7767 patients were included in each group. The incidence of postoperative liver injury was 1.4% in the sevoflurane group, which was similar to that in the propofol group (1.6%; p = 0.432). Comparison of the severity of postoperative alanine aminotransferase elevation showed that the incidence of borderline and mild elevation was higher in the sevoflurane group, but there was no difference in the incidence of moderate and severe elevation. In conclusion, sevoflurane anesthesia over 3 h was not associated with a higher incidence of clinically significant postoperative liver injury compared to propofol anesthesia.

## Introduction

Acute hepatic injury has long been considered a potential complication of general anesthesia and surgery^1–3^. Perioperative hypoxia, hypoperfusion, and hepatotoxic drugs are associated with postoperative liver injury (PLI), but the prevalence and etiology of PLI remains poorly understood. Although the impact of PLI on postoperative complications and prognosis remains unclear, multiple studies in general populations indicate that elevated serum alanine aminotransferase (ALT) levels are associated with increased morbidity and mortality^4–6^. Therefore, we believe it is important for anesthesiologists to understand the potential hepatotoxicity of the drugs they use, weigh the risks and benefits, and strive to avoid drug-induced PLI.

Halogenated inhalational anesthetics are among the most commonly used agents worldwide for maintenance of general anesthesia. Although generally considered to be safe and effective, they are known to be potentially hepatotoxic^7,8^. Halothane hepatitis, once a major drug-induced cause of acute liver failure, is believed to be caused by a hypersensitivity reaction to halothane metabolites^9^. Halothane is metabolized in the liver through cytochrome P450-mediated oxidative processes, resulting in the formation of metabolites that include trifluoroacetylate hepatic proteins^10^. These proteins have been identified as triggers for an autoimmune response that leads to PLI^11^. Modern inhalational anesthetics such as isoflurane, desflurane and especially sevoflurane undergo this metabolic process to a much lesser extent, and as a result, are less hepatotoxic^10^. Nevertheless, even with the use of these modern anesthetics, there have been documented cases of severe PLI following anesthesia, and some studies also suggest that inhalational anesthetics-induced PLI may be more common than was previously known^11–18^.

Hence, we conducted a large single-center retrospective study to determine whether using sevoflurane is a potential risk factor for PLI. The primary outcome was the incidence of PLI, defined as ALT levels elevated by more than 5 times the upper limit of the normal range (ULN) according to the American College of Gastroenterology guidelines, in patients who received either sevoflurane anesthesia or propofol-based total intravenous anesthesia (TIVA)^19^. We hypothesized that exposure to sevoflurane for more than 3 h would increase the incidence of PLI compared to propofol-based TIVA. In addition, we attempted to compare the severity of postoperative ALT elevation between the two groups.

## Methods

This was a large, single-center retrospective study approved by the Institutional Review Board of Samsung Medical Center, Seoul, South Korea (IRB no. SMC 2022-08-083) and conducted in accordance with the Declaration of Helsinki. The electronic records of adult patients who underwent general anesthesia for more than 3 h using either sevoflurane or propofol between January 2010 and September 2022 at Samsung Medical Center were reviewed. Since the data was extracted from the Clinical Data Warehouse Darwin-C of Samsung Medical Center, a system in which all the personal information of the patients is removed before medical data extraction, individual informed consents were waived by the Institutional Review Board. Patients who underwent cardiac, transplantation, or hepato-biliary-pancreatic surgeries such as liver resection and pancreaticoduodenectomy were excluded. Those with preoperative ALT values of more than 2 times the ULN (normal range of serum ALT in our institution: male 0–41 U·l^−1^; female 0–33 U·l^−1^) or no postoperative ALT values, those with missing values for the covariates used in the propensity score matching, and those in whom both sevoflurane and propofol were used for the maintenance of general anesthesia were also excluded from the study. In addition, pregnant patients, and patients with a history of general anesthesia within 21 days before or after the surgery were excluded. The following baseline patient characteristics were collected: age, sex, pregnancy, body mass index (BMI), american society of anesthesiologists (ASA) physical status, the presence of underlying diseases (alcohol consumption, smoking, chronic hepatitis B or C, liver cirrhosis, diabetes mellitus, hypertension, dyslipidemia, cerebrovascular accident, chronic obstructive pulmonary disease, coronary artery disease, congestive heart failure), and preoperative laboratory results (serum ALT, albumin, hemoglobin, and creatinine). The duration of anesthesia, type of surgery, emergency surgery, intraoperative use of vasoactive drugs (intravenous ephedrine > 5 mg or phenylephrine > 100 mcg), intraoperative vasoactive inotropic scores (VIS), the number of intraoperative packed red blood cell (RBC) or fresh frozen plasma (FFP) transfusions, and the number of hypotension episodes during surgery defined as the incidence of mean arterial pressure < 65 mmHg recorded in our electronic vital sign sheets (recorded in 5 min intervals) were extracted. Data regarding the use of common hepatotoxic drugs that are frequently used in surgical patients were extracted separately for the preoperative (defined as within 24 h before surgery), intraoperative, and the postoperative periods: steroids, acetaminophen, nonsteroidal anti-inflammatory drugs (NSAIDs), antibiotics (amoxicillin/clavulanate, isoniazid, trimethoprim/sulfamethoxazole, fluoroquinolones, macrolides, nitrofurantoin, and minocycline) and antiepileptics (phenytoin, carbamazepine, lamotrigine, and valproate)^2^. The following postoperative data were collected: serum ALT values and use of inotropes/vasopressors (dopamine, dobutamine, milrinone, norepinephrine, epinephrine, vasopressin).

The primary outcome of the study was the difference in the incidence of PLI between patients who received sevoflurane and those who received propofol-based TIVA for the maintenance of general anesthesia. A patient was classified as having PLI if any postoperative serum ALT value checked within 21 days after surgery was higher than 5 times the ULN. Since the ULN in our institution is 41 U·l^-1^ for men and 33 U·l^-1^ for women, the cut-off value for PLI was above 205 U·l^−1^ and 165 U·l^−1^ respectively. The secondary outcome was the severity of postoperative ALT elevation, as defined by the American College of Gastroenterology guidelines (maximal postoperative ALT value classified into the following categories: normal ≤ ULN; borderline ≤ 2× ULN; mild ≤ 5× ULN; moderate ≤ 15× ULN; severe > 15× ULN^19^) between the two groups.

Continuous variables such as age, BMI, preoperative laboratory results (serum ALT, albumin, hemoglobin, creatinine), duration of anesthesia, VIS, the number of intraoperative RBC and FFP transfusions, and the duration of hypotension episodes were presented as median [interquartile range] and compared using the Wilcoxon rank-sum test. Categorical variables such as sex, ASA physical status, emergency surgery, underlying diseases (alcohol consumption, smoking, chronic hepatitis B or C, liver cirrhosis, diabetes mellitus, hypertension, dyslipidemia, cerebrovascular accident, chronic obstructive pulmonary disease, coronary artery disease, congestive heart failure), intraoperative use of vasoactive agents, preoperative and postoperative medications (steroids, acetaminophen, NSAIDs, antibiotics, antiepileptics), intraoperative medications (steroids, acetaminophen, NSAIDs), postoperative use of inotropes/vasopressors, and surgical risk (classified based on the European Society of Anaesthesiology guidelines on non-cardiac surgery) were described as numbers (%) and compared using the χ^2^ test or Fisher’s exact test^20^.

To reduce bias due to confounding variables, we used propensity score matching between the sevoflurane and the propofol group. The propensity score for receiving sevoflurane (the exposure of interest) was estimated using a multivariable logistic regression model. All variables in Tables 1and 2 were used for matching, with sex as an exact matching variable since the ALT cutoff value for the diagnosis of PLI is different between males and females. A 1:1 matching with a caliper of 0.025 using the nearest neighbor method was applied. Covariate balance was assessed using standardized mean difference, with a value under 0.1 considered acceptable. After matching, the primary outcome was assessed using univariable logistic regression. The severity of postoperative ALT elevation between the two groups was compared using multinomial logistic regression. All analyses were performed using R 3.6.1 (R Development Core Team, Vienna, Austria) or SPSS (version 27, Chicago, IL, USA). A two-sided alpha of 0.05 was used for all statistical tests.

**Table 1:** Baseline characteristics in the sevoflurane and propofol groups. Values are expressed as mean (standard deviation), median [inter-quartile range], or number (%).

Variables	Overall patients				Matched patients			
	Sevoflurane (n = 31,168)	Propofol (n = 13,177)	p-value	SMD	Sevoflurane (n = 7767)	Propofol (n = 7767)	p-value	SMD
Age (year)	59.1 (13.7)	55.2 (14.0)	< 0.001	0.28	56.9 (14.7)	56.7 (14.0)	0.318	0.074
Sex, male	19,810 (63.6)	5684 (43.1)	< 0.001	0.418	3865 (49.8)	3865 (49.8)	> 0.999	< 0.001
BMI (kg/m^2^)	24.3 (3.5)	24.5 (3.7)	0.001	0.048	24.5 (3.7)	24.5 (3.6)	0.988	< 0.001
ASA physical status			< 0.001	0.081			0.518	0.029
1	7093 (22.8)	3163 (24.0)			1803 (23.2)	1836 (23.6)		
2	20,137 (64.6)	8654 (65.7)			5000 (64.4)	5035 (64.8)		
3	3729 (12.0)	1255 (9.5)			885 (11.4)	829 (10.7)		
4	195 (0.6)	97 (0.7)			74 (1.0)	63 (0.8)		
5	14 (0.0)	8 (0.1)			5 (0.1)	4 (0.1)		
Alcohol	6429 (20.6)	2787 (21.2)	0.219	0.013	1616 (20.8)	1675 (21.6)	0.255	0.019
Smoking	2709 (8.7)	1214 (9.2)	0.08	0.018	711 (9.2)	728 (9.4)	0.42	0.008
Chronic hepatitis B	1006 (3.2)	486 (3.67)	0.015	0.025	284 (3.7)	285 (3.7)	> 0.999	0.001
Chronic hepatitis C	350 (1.1)	150 (1.1)	0.927	0.001	99 (1.3)	94 (1.2)	0.772	0.006
Liver cirrhosis	426 (1.4)	91 (0.7)	< 0.001	0.067	80 (1.0)	70 (0.9)	0.46	0.013
Diabetes mellitus	4472 (14.3)	1549 (11.8)	< 0.001	0.077	1023 (13.2)	1002 (12.9)	0.634	0.008
Hypertension	9583 (30.7)	3747 (28.4)	< 0.001	0.05	2446 (31.5)	2333 (30.0)	0.052	0.032
Dyslipidemia	1717 (5.5)	989 (7.5)	< 0.001	0.081	560 (7.2)	537 (6.9)	0.491	0.012
CVA	713 (2.3)	374 (2.8)	< 0.001	0.035	225 (2.9)	219 (2.8)	0.732	0.005
COPD	975 (3.1)	181 (1.4)	< 0.001	0.118	147 (1.9)	160 (2.1)	0.489	0.012
CAD	1057 (3.4)	285 (2.2)	< 0.001	0.075	218 (2.8)	210 (2.7)	0.732	0.006
CHF	97 (0.3)	28 (0.2)	0.09	0.019	27 (0.3)	20 (0.3)	0.381	0.016
Preoperative								
Creatinine (mg/dL)	0.86 (0.42)	0.78 (0.35)	< 0.001	0.164	0.82 (0.32)	0.82 (0.42)	> 0.99	0.006
ALT (U/L)	18 [13–25]	18 [13–26]	< 0.001	0.048	18 [13–25]	18 [13–26]	0.456	0.014
Albumin (g/dL)	4.4 [4.2–4.6]	4.4 [4.2–4.6]	0.348	0.023	4.4 [4.2–4.6]	4.4 [4.2–4.6]	0.283	0.019
Hemoglobin (g/dL)	13.3 [12.3–14.4]	13.5 [12.2–14.7]	< 0.001	0.055	13.3 [12.2–14.5]	13.4 [12.3–14.5]	0.201	0.011
Steroid use	1927 (6.2)	4687 (35.6)	< 0.001	0.776	1496 (19.3)	1570 (20.2)	0.141	0.024
Acetaminophen use	1247 (4.0)	960 (7.3)	< 0.001	0.143	517 (6.7)	515 (6.6)	0.974	0.001
NSAIDs use	962 (3.1)	698 (5.3)	< 0.001	0.11	352 (4.5)	366 (4.7)	0.619	0.009
Antibiotics use	355 (1.1)	252 (1.9)	< 0.001	0.063	119 (1.5)	119 (1.5)	> 0.99	< 0.001
Antiepileptics use	185 (0.6)	709 (5.4)	< 0.001	0.284	148 (1.9)	164 (2.1)	0.391	0.015

*ASA* american society of anesthesiologists, *ALT* alanine aminotransferase, *BMI* body mass index, *CAD* coronary artery disease, *COPD* chronic obstructive pulmonary disease, *CVA* cerebrovascular accident, *NSAID* nonsteroidal anti-inflammatory drug, *SMD* standardized mean difference.

**Table 2:** Intraoperative and postoperative variables in the sevoflurane and propofol groups.

Variables	Overall patients				Matched patients			
	Sevoflurane (n = 31,168)	Propofol (n = 13,177)	p-value	SMD	Sevoflurane (n = 7767)	Propofol (n = 7767)	p-value	SMD
Emergency surgery	1443 (4.6)	841 (6.4)	< 0.001	0.077	499 (6.4)	483 (6.2)	0.621	0.008
Surgical risk			< 0.001	0.449			0.014	0.047
1	4073 (13.1)	865 (6.6)			778 (10.0)	696 (9.0)		
2	24,687 (79.2)	12,246 (92.9)			6901 (88.9)	7005 (90.2)		
3	2408 (7.7)	66 (0.5)			88 (1.1)	66 (0.9)		
Duration of anesthesia (h)	4.5 (1.6)	5.3 (1.8)	< 0.001	0.431	4.36 (0.44)	4.36 (0.40)	0.431	0.015
Vasoactive agent use	15,734 (48.7)	4,638 (33.7)	< 0.001	0.037	3114 (40.1)	3011 (38.8)	0.139	0.027
VIS	0.29 (4.03)	0.14 (1.38)	0.016	0.306	0.19 (1.61)	0.17 (1.54)	0.568	0.014
Intraoperative								
RBC (unit)	0.21 (3.69)	0.39 (3.19)	< 0.001	0.052	0.35 (4.89)	0.36 (3.86)	0.863	0.003
FFP (unit)	0.02 (0.32)	0.07 (0.70)	< 0.001	0.076	0.05 (0.46)	0.05 (0.55)	0.568	0.009
Hypotension (min)	5 [0–20]	5 [0–20]	0.988	0.004	5 [0–25]	5 [0–20]	0.185	0.023
Steroid use	2002 (6.4)	832 (6.3)	0.683	0.004	554 (7.1)	511 (6.6)	0.182	0.022
Acetaminophen use	5624 (18.0)	929 (7.1)	< 0.001	0.337	842 (10.8)	823 (10.6)	0.641	0.008
NSAIDs use	1907 (6.1)	261 (2.0)	< 0.001	0.211	227 (2.9)	218 (2.8)	0.7	0.007
Postoperative								
Steroid use	3397 (10.9)	7868 (59.7)	< 0.001	1.188	2843 (36.6)	2803 (36.1)	0.294	0.017
Acetaminophen use	16,854 (54.1)	10,003 (75.9)	< 0.001	0.47	5683 (73.2)	5600 (72.1)	0.14	0.024
NSAIDs use	9906 (31.8)	7965 (60.4)	< 0.001	0.6	3633 (46.8)	3647 (47.0)	0.834	0.004
Antibiotics use	1908 (6.1)	1544 (11.7)	< 0.001	0.185	658 (8.5)	642 (8.3)	0.664	0.007
Antiepileptics use	260 (0.8)	785 (6.0)	< 0.001	0.286	187 (2.4)	199 (2.6)	.0.871	0.01
Inotropic/vasopressor	556 (1.8)	190 (1.4)	0.012	0.027	120 (1.5)	114 (1.5)	0.742	0.006

*FFP* fresh frozen plasma, *RBC* red blood cell, *NSAID* nonsteroidal anti-inflammatory drug, *SMD* standardized mean difference, *VIS* vasoactive inotropic score.

Values are expressed as mean (standard deviation), median [inter-quartile range], or number (%). VIS = dopamine dose (mcg/kg/min) + dobutamine dose (mcg/kg/min) + 100∙epinephrine dose (mcg/kg/min) + 10∙milrinone dose (mcg/kg/min) + 10,000∙vasopressin dose (unit/kg/min) + 100∙norepinephrine dose (mcg/kg/min).

## Results

**Figure 1:**
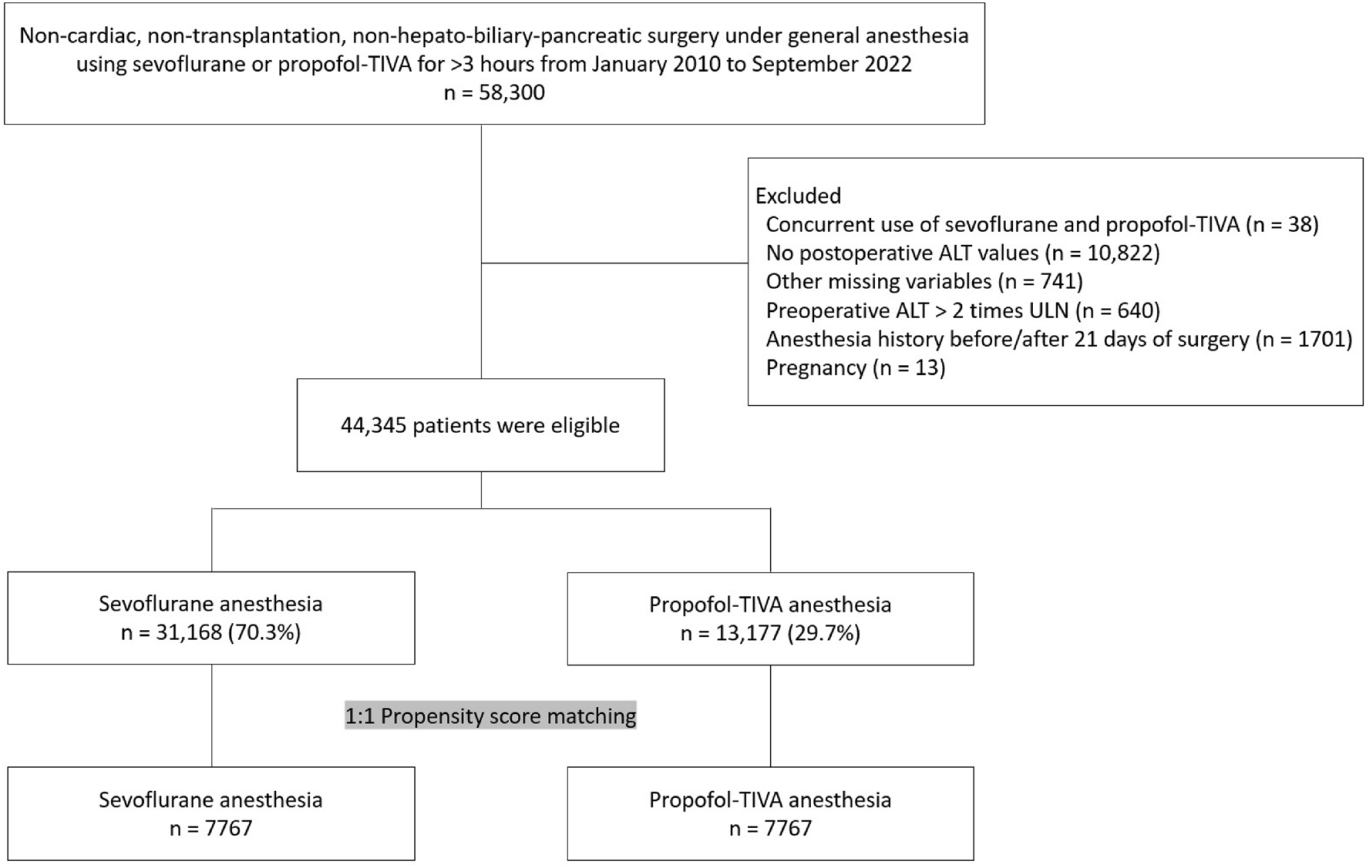
Flowchart for study population selection. *ALT* alanine aminotransferase, *TIVA* total intravenous anesthesia, *ULN* upper limit of normal range.

A total of 58,300 patients underwent non-cardiac, non-transplantation, non-hepato-biliary-pancreatic surgery under general anesthesia using sevoflurane or propofol-based TIVA for more than 3 h between January 2010 to September 2022. After excluding pregnant patients, patients who underwent general anesthesia within 21 days before or after the surgery, those who received both sevoflurane and propofol-based TIVA for maintenance of anesthesia, those with missing data, and those with preoperative ALT values higher than 2 times the ULN, 44,345 patients were eligible for the study (Fig. 1). Of these, 31,168 (70.3%) received sevoflurane while 13,177 (29.7%) received propofol for maintenance of general anesthesia. After 1:1 propensity score matching with sex as an exact matching variable, 7767 patients were included in each group. The baseline characteristics and intra/postoperative variables of the overall and matched patients are as shown in Tables 1, 2. Discrepancies of the variables between the two groups were well balanced after matching, with standardized mean differences smaller than 0.1.

Out of all the patients, 739 (1.7%) developed PLI. Among these, 501 (1.6%) were in the sevoflurane group and 238 (1.8%) were in the propofol group (p = 0*.*138). After 1:1 propensity score matching, the incidence of PLI was 1.4% (112/7767) in the sevoflurane group, which was similar to the incidence in the propofol group (1.6%, 124/7767) (p = 0.432) (Table 3).

**Table 3:** Postoperative liver injury and the characteristics of postoperative serum alanine aminotransferase elevation in the sevoflurane and propofol groups. Values are expressed as median [inter-quartile range] or number (%).

	Overall patients			Matched patients		
	Sevoflurane (n = 31,168)	Propofol (n = 13,177)	p-value	Sevoflurane (n = 7767)	Propofol (n = 7767)	p-value
Postoperative liver injury (> 5X ULN)	501 (1.6)	238 (1.8)	0.138	112 (1.4)	124 (1.6)	0.432
Severity of postoperative ALT elevation						
Normal	21,468 (68.9)	9639 (73.2)	< 0.001	5436 (70.0)	5941 (76.5)	< 0.001
Borderline elevation (≤ 2X ULN)	6794 (21.8)	2427 (18.4)	< 0.001	1687 (21.7)	1246 (16.0)	< 0.001
Mild elevation (≤ 5X ULN)	2405 (7.7)	873 (6.6)	< 0.001	532 (6.9)	456 (5.9)	0.014
Moderate elevation (≤ 15X ULN)	415 (1.3)	212 (1.6)	0.026	97 (1.3)	109 (1.4)	0.44
Severe elevation (> 15X ULN)	86 (0.3)	26 (0.2)	0.124	15 (0.2)	15 (0.2)	> 0.99
Time to maximal ALT value (days)	2 [0–7]	4 [1–7]	< 0.001	3 [1–7]	3 [0–7]	0.027

*ALT* alanine aminotransferase, *ULN* upper limit of the normal range.

**Figure 2:**
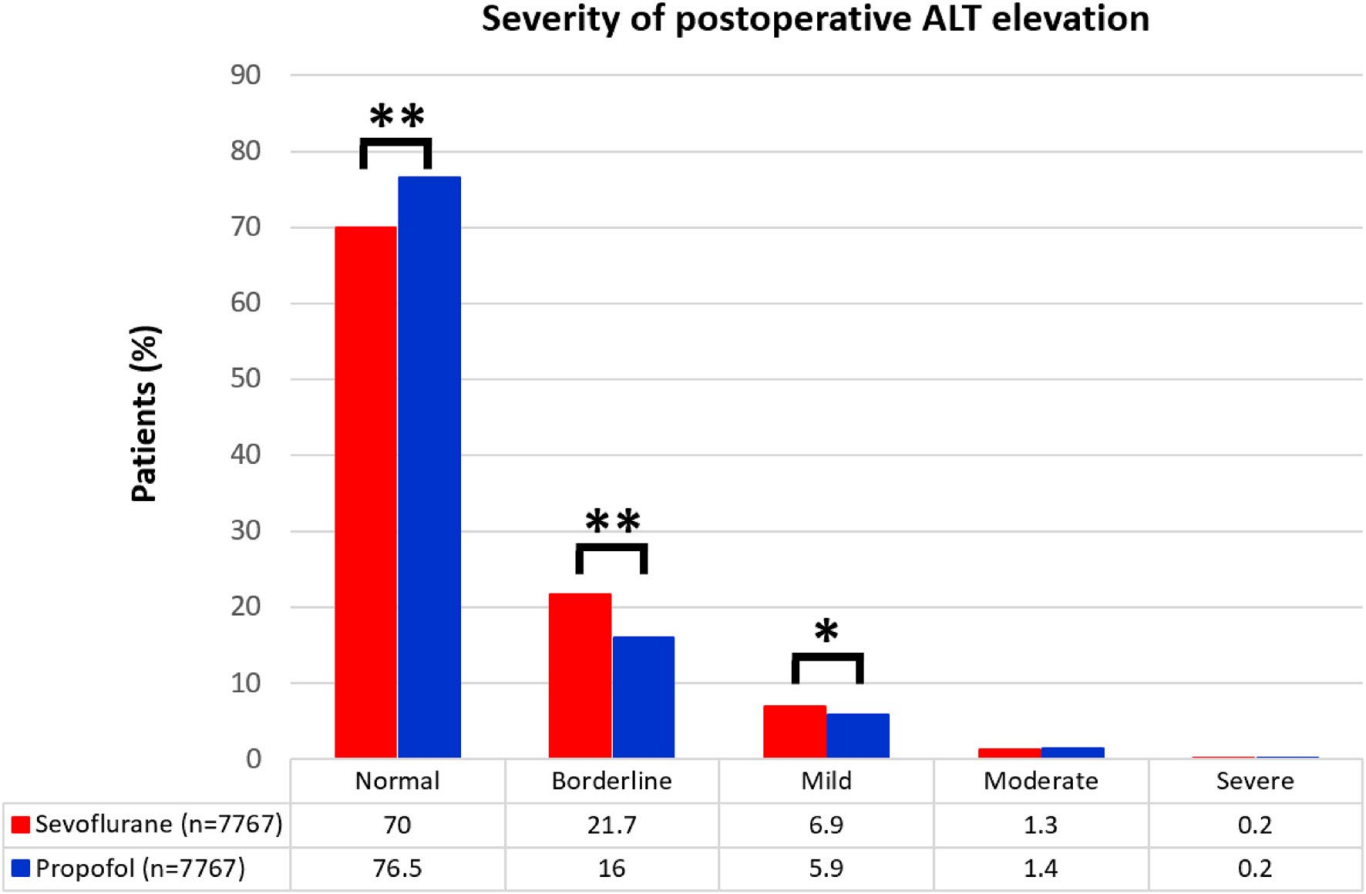
Comparison of the severity of postoperative ALT elevation between the sevoflurane and the propofol group. *ALT* alanine aminotransferase. *p < 0.05; **p < 0.001.

Comparison of the severity of postoperative ALT elevation between the two groups revealed that using sevoflurane was associated with a higher risk of developing borderline or mild elevation than using propofol (OR 1.480; 95% CI 1.363–1.606; p < 0.001 and OR 1.275; 95% CI 1.119–1.453; p < 0.001, respectively). However, there were no significant differences between the two groups in terms of patients with moderate or severe elevations (OR 0.973; 95% CI 0.738–1.282; p = 0.844 and OR1.093; 95% CI 0.534–2.238; p = 0.808, respectively) (Table 3 , Fig. 2).

**Figure 3:**
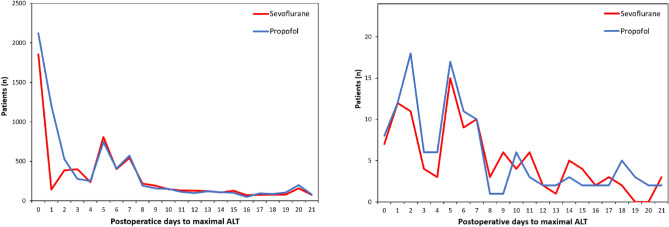
The time to maximal ALT value within postoperative 21 days in the propensity score-matched cohort (left) and in patients with PLI (right). *ALT* alanine aminotransferase, *PLI* postoperative liver injury.

In the matched cohort, the maximal postoperative ALT values were primarily observed within POD 7. For patients who developed PLI, the maximal ALT values were distributed with a double peak of similar size at POD 0 to 2 and POD 5 to 7 (Fig. 3).

## Discussion

In this study, using sevoflurane for maintenance of general anesthesia was not associated with PLI when compared with using propofol. Although an increased risk of borderline and mild elevation (1× to 5× ULN) of postoperative ALT values was observed in the sevoflurane group, the incidence of moderate and severe ALT elevation (> 5× ULN) did not differ significantly between the two groups. These findings suggest that the use of sevoflurane is unlikely to exhibit clinically significant hepatotoxic properties.

There are various factors that could contribute to PLI. Hypoperfusion of the liver is one of the common causes of postoperative ALT elevation^21^. Also, certain classes of antibiotics, antiepileptics and NSAIDs are well-known for their hepatotoxic properties^2,3^. Therefore, we included them as variables in our propensity score matching process, thereby ensuring balance between the sevoflurane and propofol groups.

A study by Oh et al. demonstrated that the use of sevoflurane for maintenance of anesthesia resulted in higher postoperative liver enzyme values compared to the use of propofol in patients with elevated preoperative serum liver enzymes^15^. However, in the study, some important factors that may cause PLI such as the use of hepatotoxic drugs in the postoperative period were not considered. Also, ALT values only within 72 h after surgery were collected. In our study, ALT values up to 21 days postoperatively were collected since PLI due to sevoflurane usually results in ALT elevation within 2 to 21 days after surgery^11,22^.

Two small studies investigated the incidence of PLI in trauma and surgical patients after the administration of modern inhalational anesthetics^12,16^. To identify possible inhalational anesthetics-induced PLI, these studies used the Council for International Organizations of Medical Sciences / Roussel Uclaf Causality Assessment Method (CIOMS/RUCAM) scoring system, which has been validated as a method of determining the likelihood of a drug as the causative agent of PLI. The studies reported that the incidence of PLI possibly caused by inhalational anesthetics was between 3 and 4.1%, which is somewhat higher than the results of our study. Although the CIOMS/RUCAM score was used to rule out inhalational anesthetics-irrelevant cases of PLI, it may have been insufficient to completely exclude them. Also, in these studies, the authors used a rather broad CIOMS/RUCAM score range for the diagnosis of possible inhalational anesthetics-induced PLI. Therefore, true inhalational anesthetics-induced PLI cases may be less than what these two studies have reported.

We considered it reasonable to compare sevoflurane with propofol to assess the potential hepatotoxicity of sevoflurane. Although propofol infusion syndrome, a potentially fatal complication that may occur after prolonged administration of high doses (typically exceeding 48 h at > 5 mg·kg^−1^·hr ^−1^), is a known cause of acute liver injury, propofol is generally considered safe in terms of hepatotoxicity when administered in conventional doses during surgery^23,24^. Moreover, research has demonstrated that patients with pre-existing liver diseases can safely receive TIVA with propofol^25^.

We used serum ALT values for the diagnosis of PLI because inhalational anesthetics-induced PLI is known to be mainly hepatocellular, which is characterized by a rise in serum ALT levels^11,22,26^. The ALT value threshold for PLI in this study was determined based on the definitions of ALT elevation in the American College of Gastroenterology guidelines. According to the guidelines, ALT values higher than 5 times the ULN are advised to undergo immediate evaluation of the liver^2,19^.

For patients who developed PLI, the time point of maximal postoperative ALT values showed a double-peak distribution in both groups. Since PLI due to hepatic ischemia typically results in an immediate increase in serum ALT value that peaks within 48 h, the first peak is likely to be mainly due to liver hypoperfusion during or immediately after surgery^21^. In contrast, the second peak may have been caused by the hepatotoxicity of intraoperative or postoperative drugs including anesthetics. The majority of maximal ALT values were observed within 7 days after surgery, and therefore patients with a higher risk of developing PLI may benefit from closer monitoring during this period.

This study has several limitations. First, because of its retrospective design, the data may have been biased or inaccurate. We attempted to minimize these risks by using large data and performing propensity score matching with a small caliper. However, since liver enzyme elevation can be caused by various factors, there may have been hidden factors missed out in the propensity scoring. Second, we categorized all surgical procedures as low, intermediate, or high surgical risk, but a more detailed categorization may have reduced bias. Although we excluded surgeries that can directly injure the liver, it is still possible that procedures involving manipulation of the liver or alteration of hepatic blood flow were included. These procedures could potentially act as confounding variables when assessing anesthesia-related PLI. Third, since preoperative serum ALT value was defined as the most recent value within 6 months before surgery, some patients may have had undiagnosed liver injury that developed during the time interval between the last serum ALT test and surgery. Finally, postoperative ALT values were checked not daily, but depending on the physician’s decision. Therefore, it is necessary to consider this aspect when interpreting the postoperative trends of ALT changes. However, since tracking ALT values daily for an extended period after surgery is impractical in real clinical practice, we believe that the results of our analysis may provide useful information for clinicians to reference.

In conclusion, our study suggests that sevoflurane anesthesia over 3 h has minimal hepatotoxic properties comparable to propofol-based TIVA. Prospective studies are needed to confirm our findings, and further research is necessary to determine long-term prognosis.

## Data Availability

The datasets generated and analyzed during the current study are available from the corresponding author on reasonable request.
